# Serum IgA antibody level against porcine epidemic diarrhea virus is a potential pre-evaluation indicator of immunization effects in sows during parturition under field conditions

**DOI:** 10.1186/s40813-024-00382-w

**Published:** 2024-09-03

**Authors:** Zhiqiang Hu, Yang Li, Bingzhou Zhang, Ying Zhao, Ran Guan, Yapeng Zhou, Jiafa Du, Zhimin Zhang, Xiaowen Li

**Affiliations:** 1 College of Animal Science, Xichang University, No.1 Xuefu Road, Anning Town, Xichang, 615013 Sichuan Province P. R. China; 2 Shandong Engineering Research Center of Pig and Poultry Health Breeding and Important Infectious Disease Purification, Shandong New Hope Liuhe Group Co., Ltd, No. 592-26 Jiushui East Road Laoshan District, 266100 Qingdao, Shandong, P. R. China

**Keywords:** Porcine Epidemic Diarrhea virus, Serum/colostrum, IgA/IgG, Neutralizing antibodies, Evaluation indicator

## Abstract

**Background:**

Porcine Epidemic Diarrhea (PED) is a highly contagious disease caused by Porcine Epidemic Diarrhea Virus (PEDV), resulting in a mortality rate of suckling piglets as high as 100%. Vaccination is the primary strategy for controlling PEDV infection, however, there is currently a lack of reliable methods for assessing the efficacy of vaccination. This study aimed to analyze serum and colostrum samples from 75 parturient sows with a specific vaccination strategy to measure levels of IgG, IgA, and neutralizing antibodies (nAbs) against PEDV, and to investigate the correlation between serum and colostrum antibody levels, as well as to identify potential biomarkers that can be used to evaluate immunization effects under field conditions.

**Results:**

The findings of correlation analysis between antibody levels of IgA, IgG, and nAbs in serum or colostrum samples revealed that IgG demonstrated the most robust correlation with nAbs exhibiting a correlation coefficient of 0.64 in serum samples. Conversely, IgA exhibited the highest correlation with nAbs, with a correlation coefficient of 0.47 in colostrum samples. Additionally, the correlation analysis of antibody levels between serum and colostrum samples indicated that serum IgA displayed the strongest correlation with colostrum IgA, with a coefficient of 0.63, indicating that serum IgA may serve as a viable alternative indicator for evaluating IgA levels in colostrum samples. To further evaluate the suitability of serum IgA as a substitute marker for colostrum IgA, levels of IgA antibodies in serum samples from sows were examined both pre- and post-parturition. The findings indicated that serum IgA levels were initially low prior to the initial immunization, experienced a notable rise 21 days after immunization, and maintained a significant elevation compared to pre-immunization levels from 21 days pre-parturition to 14 days postpartum, spanning a total of 35 days.

**Conclusions:**

Serum anti-PEDV IgA antibody levels may serve as a valuable predictor for immunization effects, allowing for the assessment of colostrum IgA antibody levels up to 21 days in advance. This insight could enable veterinarians to timely adjust or optimize immunization strategies prior to parturition, thereby ensuring adequate passive immunity is conferred to piglets through colostral transfer postpartum.

## Background

Porcine Epidemic Diarrhea (PED) is a highly contagious disease caused by the Porcine Epidemic Diarrhea virus (PEDV), leading to symptoms such as elevated body temperatures, anorexia, vomiting, severe diarrhea, and dehydration in swine of all ages [1, 2]. Since the initial outbreak of PEDV in China in 2010, the virus has led to a mortality rate of suckling piglets as high as 100%. This disease annually causes significant financial losses amounting to billions of yuan in the swine industry, making PEDV the main pathogen responsible for swine viral diarrhea and the second most significant threat to the industry after African swine fever virus (ASFV) [3, 4]. PEDV is a single-stranded positive-sense RNA virus classified in the *Alphacoronavirus* genus of the *Coronaviridae* family. The genome of PEDV is 28 kb long and contains seven open reading frames (ORFs), which encode two large polyproteins (pp1a and pp1b), four structural proteins (spike, membrane, envelope, and nucleocapsid proteins), and one accessory protein (ORF3) [5]. Due to the rise in epidemic PEDV strains and immune pressure from vaccine strains, PEDV has been evolving and mutating through genetic changes such as insertions, deletions, and recombination [6]. This has resulted in the development of new branches, subtypes, and genotypes.

The pig farming model in China is shifting towards more intensive large-scale sow farms with a population of 1000 or more sows due to the impact of ASFV. As a result, immunization by vaccination is the primary method for controlling PEDV infection. Large-scale farms are facing challenges in selecting effective vaccines and developing optimal immunization strategies. Our previous study indicates that immunizing sows with the PEDV NH-2020 strain followed by a second dose of commercial inactivated vaccines is more effective than certain commercial vaccines alone [6, 7]. This method offers improved protection for suckling piglets, reducing the probability of PEDV outbreaks [7]. Nevertheless, due to the extensive workload of vaccine immunization in large-scale pig farms, batch immunization processes may result in inconsistent post-immunization effects. Additionally, there is currently no reliable method for evaluating the effectiveness of immunization.

Vaccination or re-feeding adult pigs triggers the production of neutralizing antibodies (nAbs), PEDV-specific IgG and IgA antibodies in serum within 7–14 days, aiding in clearing PEDV in sows [8, 9]. Due to the slow regeneration of intestinal villi epithelial cells and the underdeveloped immune system in young piglets [8], ensuring adequate protection for suckling piglets is critical for effective controlments against PEDV in intensive farming environments. The protection of suckling piglets relies on the levels of antibodies in colostrum and milk [10]. It has been reported that nAbs in colostrum and milk are related to maternal secreted IgA (sIg A) antibodies [11, 12]. PEDV-specific sIgA antibodies cannot be produced by mammary glands of female sows due to the absence of lymphoid follicles in mammary glands. When pregnant sows are vaccinated against PEDV, the intestinal mucosal immune response is activated, and these antibodies can be synthesized by effector B cells or plasmablasts, which migrate from the gut-associated lymphoid tissue of PEDV-infected intestines to the mammary glands [12, 13]. Through the gut-mammary sIgA axis, these antibodies are subsequently secreted into colostrum and milk. Upon ingestion by piglets, the IgA in the colostrum and milk adheres to the intestinal mucosa, thereby providing localized immune protection. Therefore, the level of sIgA antibodies in colostrum and milk is crucial and has been used widely for evaluating the extent of passive immunity against PEDV infection in piglets [8]. However, the timing of collecting colostrum samples in clinical settings after the sow gives birth delays test results, making it difficult for veterinarians to assess immunization effects on piglets in advance. Despite veterinarians obtaining accurate assessments of immunization effects on piglet protection through colostrum or milk samples post-parturition, there are no effective measures to prevent the occurrence of epidemics at this time under field conditions currently. Additionally, the collection of colostrum during sow delivery in large-scale farming operations is expected to significantly increase the workload for staff. The serum sample represents a significant option for testing antigens and antibodies of various swine pathogens under field conditions, with the advantage of no timing constraints for its collection. However, the correlation between anti-PEDV antibodies in serum and colostrum samples remains uncertain.

This study involved the collection of serum and colostrum samples from 75 parturient sows with a specific immunization strategy against PEDV. The primary objective was to quantify the levels of IgG, IgA, and nAbs and to evaluate the correlations between these antibody indicators in serum and colostrum samples. The goal was to identify an earlier indicator and a more easily collectible sample type that could serve as a substitute for colostrum in assessing immunization effects under field conditions. The results are expected to provide valuable insights for evaluating the efficacy of PEDV immunization and for optimizing immunization strategies under field conditions.

## Method

### Farm description

**Fig. 1:**
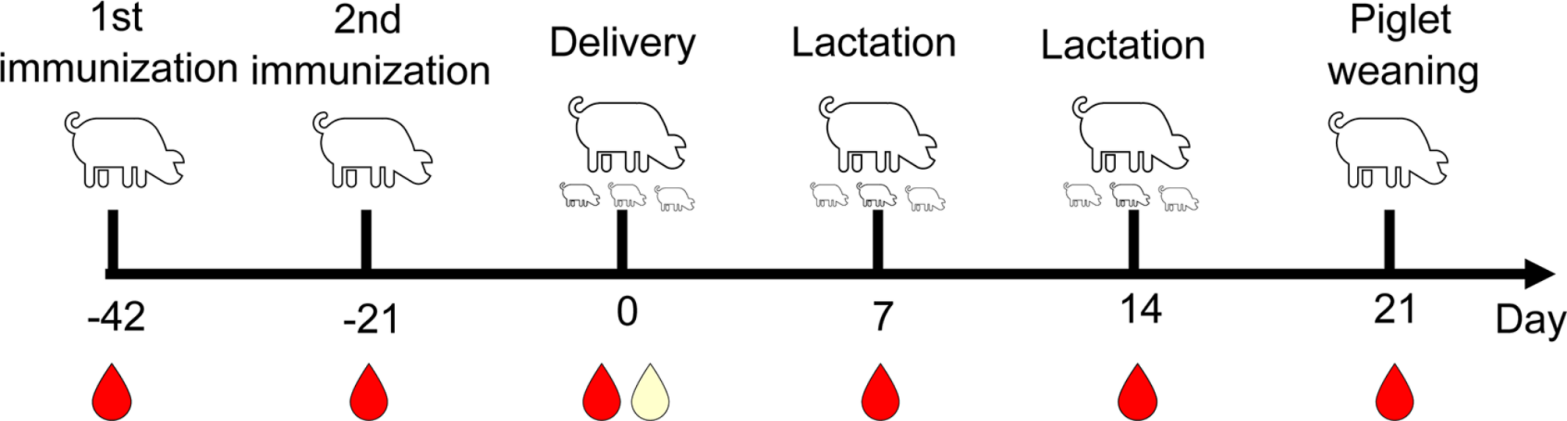
The timeline of immunization and sample collection

This study focused on a commercial breeding farm in Gansu Province, China, which operated on a scale of 1,500 sows (Large-White × Landrace). According to the production performance of this farm in 2023, the average number of born and weaned piglets per litter is 12.1 and 10.5, respectively. The farm comprises one breeding house and five farrowing houses, each with a capacity to accommodate 60 sows. The farrowing schedule is organized in batches, with each batch consisting of 300 sows. The immunization strategy against PEDV for the farrowing sows is detailed as follows: all sows were initially vaccinated with a highly virulent live vaccine strain (NH-TA2020) [6], isolated by Swine Research Institute of New Hope Group, administered orally 42 days prior to delivery. Subsequently, the sows received a commercial inactivated vaccine (AJ1102 strains, Wuhan Keqian Biology Co., Ltd) 21 days before delivery. The immunization timeline was depicted in Fig. 1. Specifically, sows scheduled for delivery were required to fast for 12 h prior to the first vaccination. Subsequently, 3 mL of the vaccine solution was mixed with 1.5 kg of feed to ensure complete consumption by the sows. After a period of 21 days, a commercially available vaccine was administered via intramuscular injection in the neck, with a dosage of 2 mL per sow.

### Sample collection

Seventy-five sows were randomly selected, with fifteen sows chosen from each farrowing house. As illustrated in Fig. 1, blood samples were obtained from the anterior vena cava of individual sows on the 42nd and 21st days prior to parturition (pre-vaccination), on the day of parturition, and on the 7th, 14th, and 21st days postpartum (the day the piglets were weaned), with each sample consisting of 10 mL per sow. Subsequently, serum was separated from the blood samples by centrifugation, yielding 3–5 mL per sample. Colostrum samples were collected within 6 h postpartum, ensuring that the newborn piglets could suckle. Sows were not restrained during the collection process, which was conducted in the presence of the piglets. Colostrum was obtained from the first three pairs of teats, from head to tail, with a volume of 1.5 -2 mL each teat and pooled per sow; in total approximately 10 mL per sow.

### Detection of antibody levels of PEDV-IgA and IgG

The antibody levels of PEDV-IgA in serum and colostrum were quantified using commercial ELISA kits (Cat: 06-55550-01, IDEXX Laboratories, Westbrook, ME, USA), following the manufacturer’s protocol. Absorbance values were measured and recorded at 650 nm using a microplate reader (Multiskan FC, Thermo Fisher Scientific, Waltham, MA, USA). S/P value = (Sample-NC)/(PC-NC). NC: negative control; PC: positive control. S/*P* ≥ 0.50 was considered as positive, S/*P*<0.50 was regarded as negative.

The antibody levels of PEDV-IgG in serum and colostrum were quantified using commercial ELISA kits (Cat: 23100205-S, Combetter Biotechnology Co., Ltd., Hunan, P. R. China), following the manufacturer’s protocol. Absorbance values were measured and recorded at 450 nm using a microplate reader (Multiskan FC, Thermo Fisher Scientific, Waltham, MA, USA). S/P value= (Sample-NC)/(PC-NC). S/*P* ≥ 0.40 was considered as positive, S/*P*<0.40 was regarded as negative.

### Detection of neutralizing antibody titers

Serum and colostrum samples were initially inactivated by incubation at 56 °C for 30 min. Subsequently, a penicillin-streptomycin-amphotericin B solution was introduced to the inactivated samples. The colostrum or serum samples were then subjected to serial dilutions at ratios of 1:10, 1:20, 1:40, 1:80, 1:160, 1:320, 1:640, 1:1280, 1:2560, 1:5120, and 1:10,240. Each dilution was taken for 250 µL and combined with 250 µL of PEDV inoculum (200 50% tissue culture infective dose [TCID50]/0.1 mL). After the mixture incubated at 37 °C for 1 h, 100 µL was taken and transferred to 96-well plates containing a monolayer of Vero cells and incubated at 37 °C for 1 h. Following this incubation, Dulbecco’s Modified Eagle Medium (DMEM) supplemented with trypsin (5 mg/mL) was added to each well. The plates were then incubated at 37 °C for a period ranging from 3 to 5 days. Each sample was replicated four times, and the cytopathic effects (CPEs) were monitored daily [14]. Titers of nAbs were quantified and expressed as the log10 transformation of the highest reciprocal serum or colostrum dilution capable of protecting 50% of tissue culture cells from lesions.

### Statistical analysis

Pearson correlation analysis between different samples was conducted using the GraphPad Prism software (version 8.0), with correlation coefficients represented by r values and statistical significance defined as *p* values less than 0.05. Additionally, the mean IgA antibody levels among different samples were compared using the paired t-test in the GraphPad Prism software (version 8.0) as well. A *p* value of < 0.05 was considered to be statistically significant.

## Results

### Correlation analysis between antibody levels of IgA, IgG and nAbs in either serum or colostrum samples

**Fig. 2:**
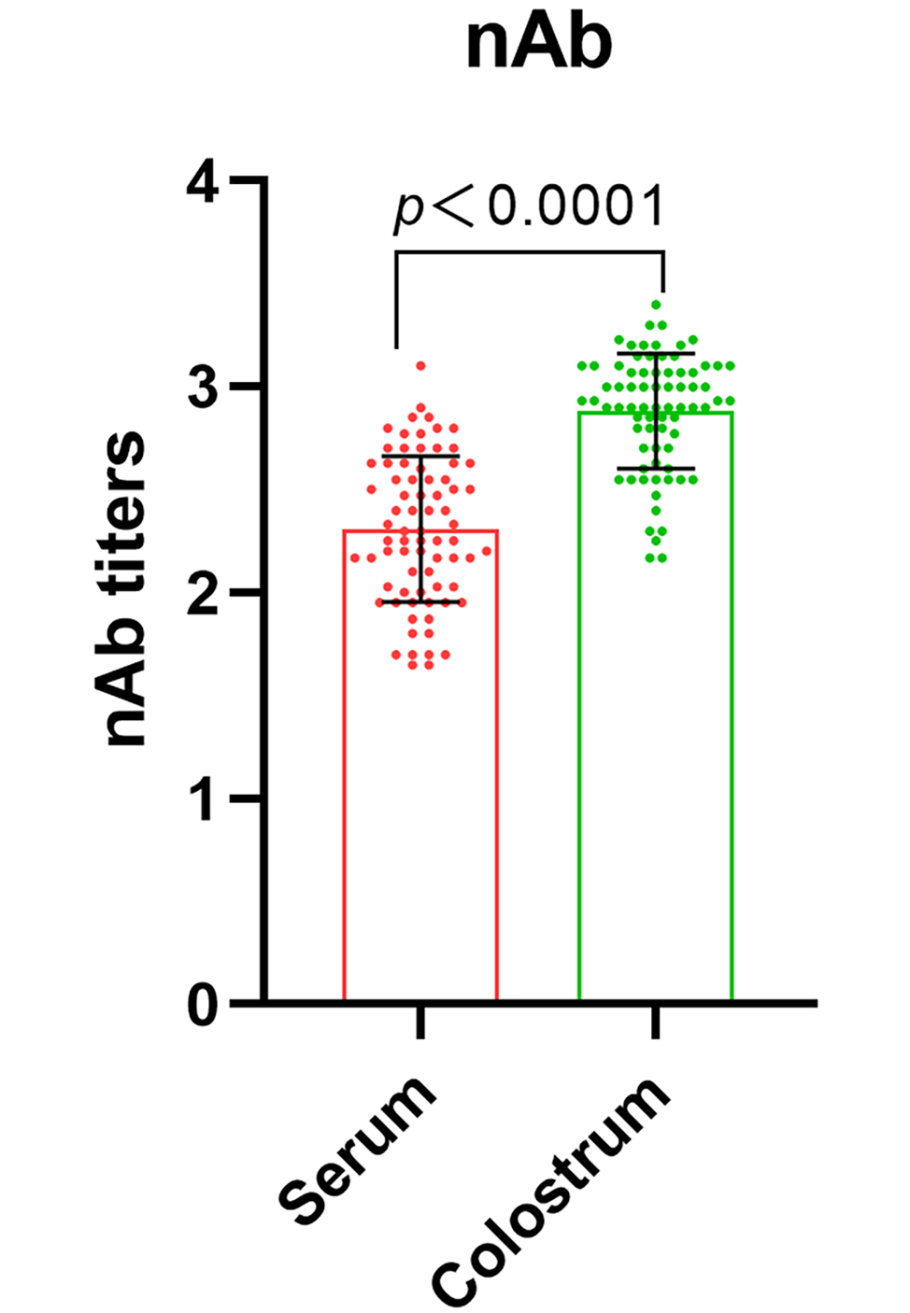
nAb titers in colostrum and serum samples

**Fig. 3:**
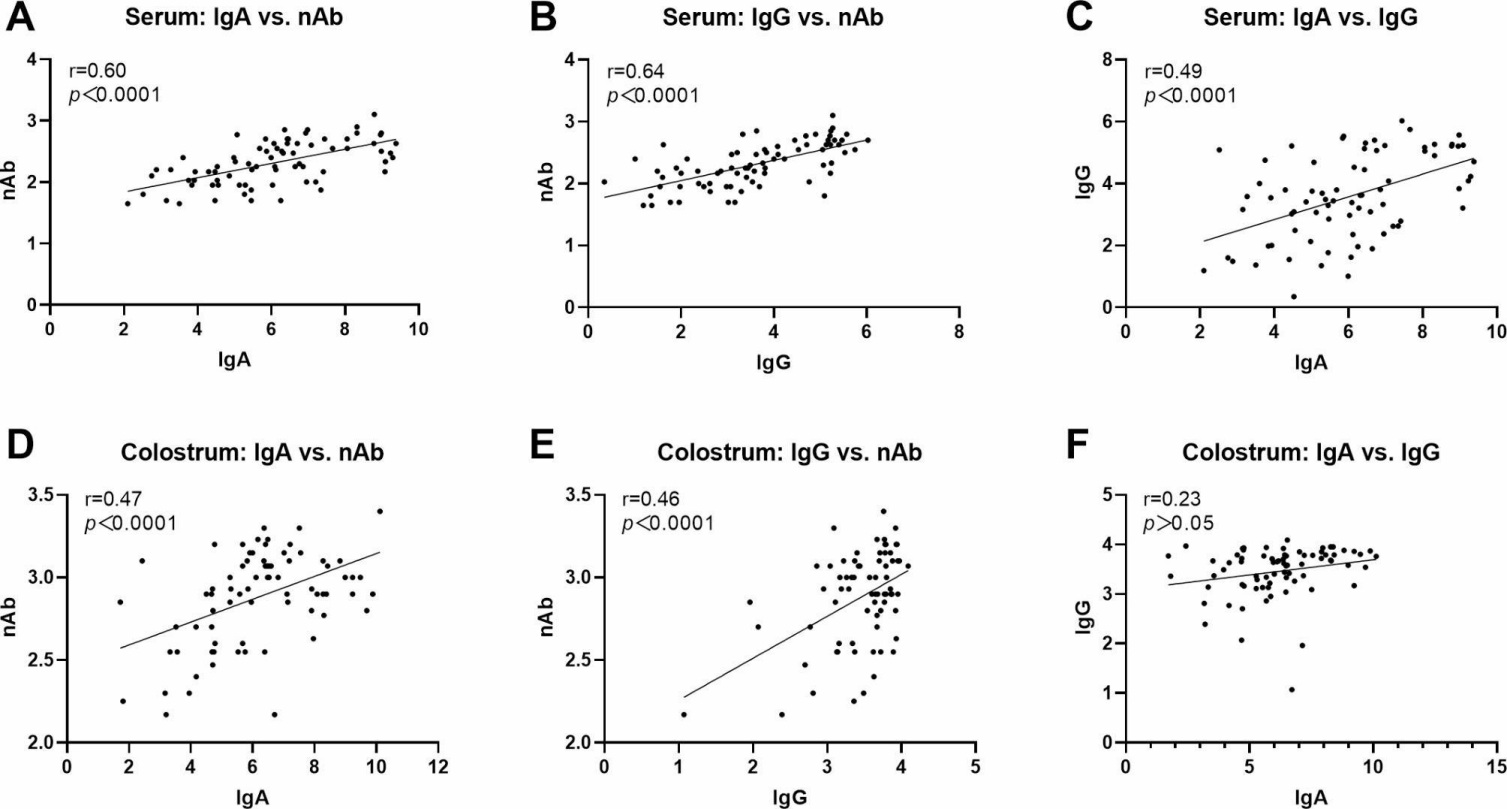
Correlation analysis between the antibody levels of IgA, IgG and nAb in either serum (**A-C**) or colostrum samples (**D-F**). **A** and **D**: IgA vs. nAb; **B** and **E**: IgG vs. nAb; **C** and **F**: IgG vs. IgA. “r” values represented correlation coefficients. “*p*” values meant statistical significances

The results of nAb titers from 75 sows showed that colostrum samples were with higher levels of antibodies compared to serum on the day of parturition, as illustrated in Fig. 2. This indicates that the immunization strategy successfully boosted antibody levels in colostrum, offering protection to suckling piglets. We further analyzed relationships between various antibody indicators in serum and colostrum by conducting correlation analyses of IgA, IgG, and nAbs. In serum samples (Fig. 3A, B **and C**), IgG exhibited the strongest correlation with nAbs, with a correlation coefficient of 0.64 (*p* < 0.0001), followed by IgA at 0.60 (*p* < 0.0001). Additionally, IgA and IgG showed a significant correlation with a coefficient of 0.49 (*p* < 0.0001). In colostrum samples (Fig. 3D, E **and F**), IgA displayed the highest correlation with nAbs at 0.47, followed by IgG at 0.46 (*p* < 0.0001). There was no significant correlation between IgG and IgA in colostrum (*p* > 0.05).

### Correlation analysis of antibody levels of IgA, IgG and nAbs between serum and colostrum samples

**Fig. 4:**
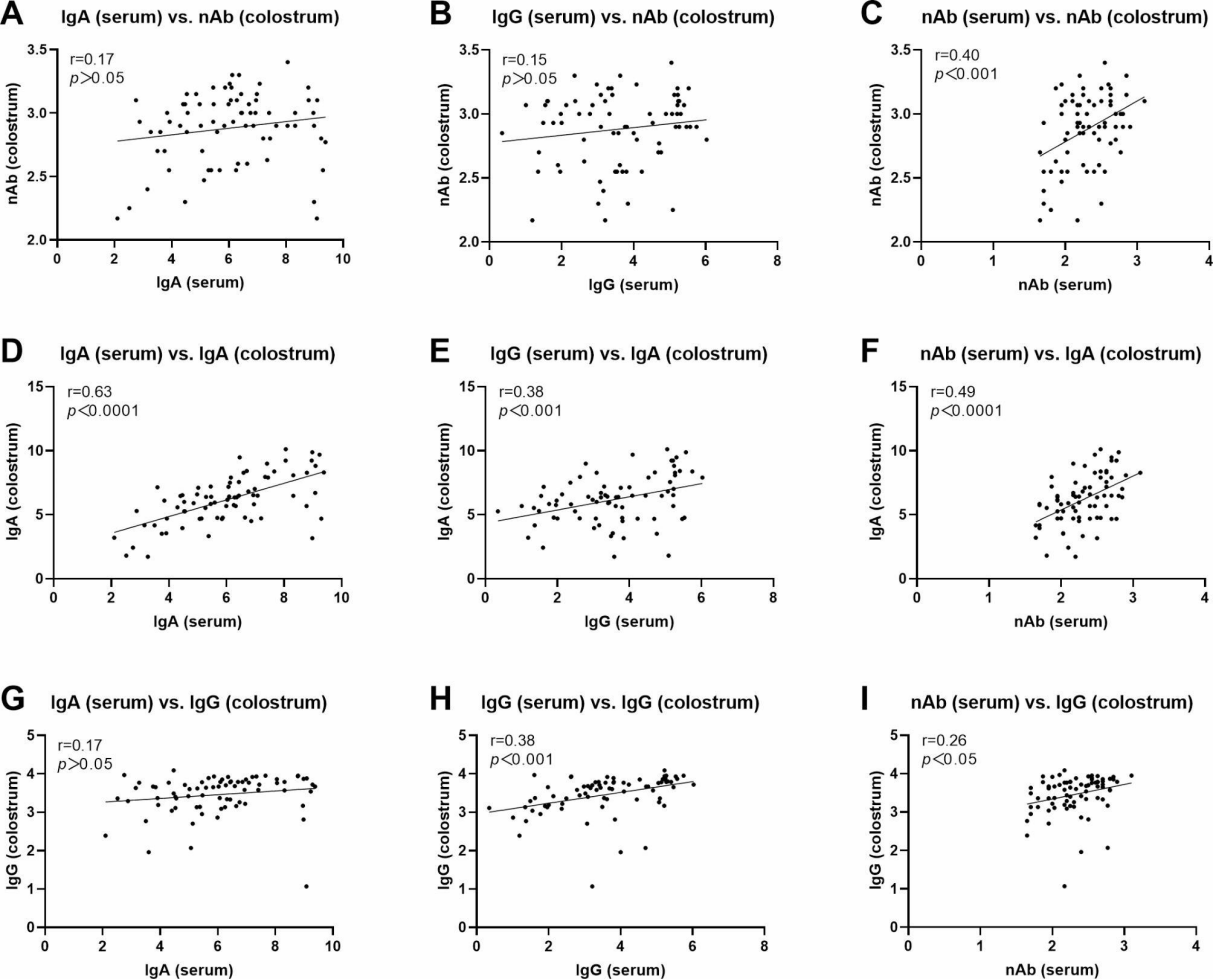
Correlation analysis of the antibody levels of IgA, IgG and nAb between serum and colostrum samples. **A-C**: Correlations of nAbs in colostrum samples with IgA (**A**), IgG (**B**) and nAbs (**C**) in serum samples; **D-F**: Correlations of IgA in colostrum samples with IgA (**D**), IgG (**E**) and nAbs (**F**) in serum samples; **G-I**: Correlations of IgG in colostrum samples with IgA (**G**), IgG (**H**) and nAbs (**I**) in serum samples. “r” values represented correlation coefficients. “*p*” values meant statistical significances

Further analyses of relationships between antibody indicators in serum and colostrum samples revealed that serum nAbs exhibited a significant correlation with those in colostrum samples, as evidenced by a correlation coefficient of 0.40 (*p* < 0.001) in Fig. 4C. However, serum IgA or IgG did not demonstrate a significant correlation with colostrum nAbs (*p* > 0.05) (Fig. 4A **and B**). Notably, serum IgA displayed the strongest correlation with colostrum IgA, with a coefficient of 0.63 (*p* < 0.0001) (Fig. 4D), indicating that serum IgA may serve as a viable alternative indicator for evaluating IgA levels in colostrum samples. Serum nAbs exhibited a significant correlation with colostrum IgA, as evidenced by a correlation coefficient of 0.49 (*p* < 0.0001) (Fig. 4F). Similarly, serum IgG demonstrated a moderate correlation with colostrum IgA, albeit with a lower coefficient of 0.38 (*p* < 0.001) (Fig. 4E). In comparison to colostrum IgG, both serum IgG and nAbs displayed a weaker positive correlation with colostrum IgG levels, with respective coefficients of 0.38 (*p* < 0.001) (Fig. 4H) and 0.26 (*p* < 0.05) (Fig. 4I). Notably, there was no significant correlation observed between serum IgG and colostrum IgG levels (*p* > 0.05) (Fig. 4G).

### Changes of IgA antibody levels before and after parturition

**Fig. 5:**
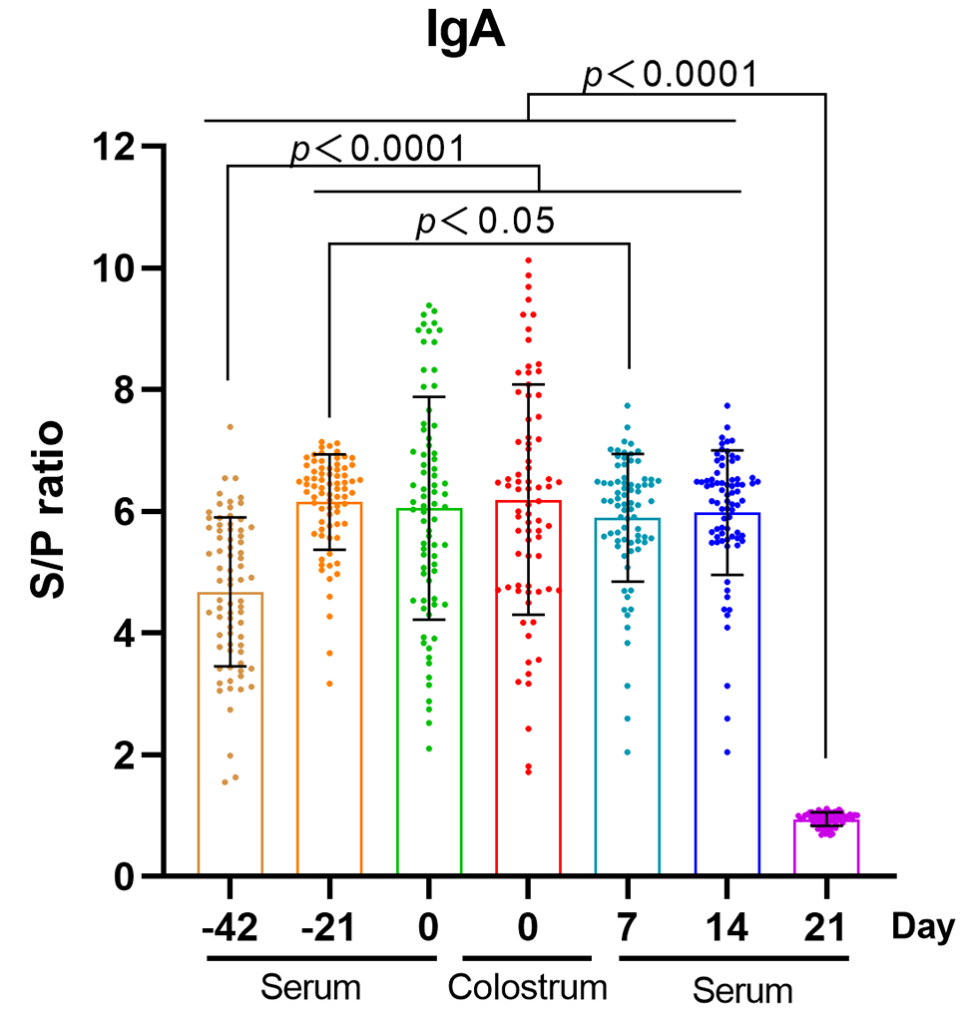
Changes of IgA antibody levels before and after parturition. A *p* value of < 0.05 was considered to be statistically significant

To further assess the potential of serum IgA as an alternative marker for colostrum IgA, we measured IgA antibody levels in serum samples from sows collected on the 42nd and 21st days prepartum, on the day of parturition, and on the 7th, 14th, and 21st days postpartum. These measurements were then compared with colostrum IgA levels at parturition. As illustrated in Fig. 5, serum IgA antibody levels were initially low prior to the first immunization, exhibited a significant increase 21 days post-immunization (*p* < 0.0001), and remained significantly elevated compared to pre-immunization levels from the 21st day prepartum to the 14th day postpartum. Except for a significant decrease in antibody levels observed on the 7th day postpartum compared to the 21st day prepartum (*p* < 0.05), no significant differences were detected among the other groups, which included the 21st day prepartum, the day of parturition, the 7th or the 14th days postpartum (*p* > 0.05). Furthermore, colostrum IgA levels on the day of parturition did not significantly differ from serum IgA levels in the other groups, including the 21st day prepartum, the day of parturition, the 7th or the 14th days postpartum (*p* > 0.05). Notably, antibody levels reached their lowest point on day 21 postpartum. These findings suggest that, under the current immunization strategy, serum IgA levels begin to stabilize at a high level from 21 days prepartum and remain elevated for a duration of 35 days. Consequently, serum IgA levels can be monitored starting 21 days before parturition to evaluate the immunization efficacy in the current batch of pigs. For pigs exhibiting low serum IgA levels, booster immunizations may be administered to mitigate the risk of instability in the PEDV epidemic status within the parturient pig population, which could arise from individual pigs missing initial immunizations.

## Discussion

Immunization of sows with PEDV vaccines or prior exposure to PEDV can induce lactogenic immunity, which is subsequently transferred to piglets through maternal colostrum and milk [15–17]. Suckling piglets that acquire colostrum antibodies exhibit markedly reduced PEDV RNA loads in their fecal samples, thereby receiving enhanced protection against the virus [16, 17]. Findings of Bourne and Curtis suggest that nearly 100% of IgG, 40% of IgA, and 85% of IgM in colostrum are derived from serum, whereas approximately 70% of IgG and 90% of IgA and IgM in milk are synthesized locally within the mammary glands [18]. In this study, we found that nAb titers in colostrum were significantly higher than those in serum (Fig. 2), which is consistent with the results of Qinye Son et al. [10]. This may be due to the transfer of large amounts of anti-PEDV antibodies from the serum to colostrum during sow parturition. Further literatures indicate that colostrum antibodies are predominantly composed of IgG, IgA, and IgM, with IgG being the most prominent. These antibodies can be absorbed by piglets and enter the bloodstream, thereby providing systemic protection. In contrast, IgA immunoglobulins adhere to the intestinal lining, offering localized immunity for piglets [19, 20]. In this study, the correlation coefficient between the levels of nAbs and IgA antibodies in colostrum was found to be the highest, corroborating the finding of Song et al. [21]. And, the correlation coefficient between IgG and nAbs was very close to that between IgA and nAbs. These results suggest that colostrum may confer protective effects against PEDV in suckling piglets through both IgG and IgA antibodies during early postpartum lactation. Similar observations have been reported in the context of transmissible gastroenteritis virus (TGEV) infections [12].

In intensive sow herds, monitoring pathogen and antibody levels is essential for implementing effective measures to control PEDV infection in sows [22]. Although substantial literature suggests that IgA levels in colostrum or milk are inversely related to piglet mortality and can serve as indicators for assessing the protective immunity of piglets [8, 11–13], this reliance on colostrum or milk introduces a delay in obtaining results and hinders the timely monitoring and assessment of the efficacy of prepartum vaccination. Interestingly, some studies demonstrate that high levels of IgG in serum are induced when pigs are immunized with either attenuated or inactivated PEDV, while there is no corresponding on serum IgA levels. Furthermore, subsequent challenge experiments indicate that this method does not confer complete protection in piglets. In contrast, exposure to a highly virulent strain of PEDV elicits robust levels of both IgG and IgA antibodies in serum, which can provide full protection for subsequent challenge piglets [12, 23]. Our prior clinical studies have also demonstrated that the strategy involving virulent PEDV is superior to that of commercial attenuated PEDV vaccines [7]. In this study, employing the strategy of oral vaccination with virulent PEDV strain and intramuscular vaccination with a commercial inactivated vaccine, we identified the highest correlation between serum IgA and colostrum IgA antibody levels at parturition (*r* = 0.63). Research conducted by Gerber PF and Opriessnig T also corroborates a positive correlation between serum IgA levels and fecal IgA levels, another important indicator of mucosal immunity against PEDV [24]. This underscores the potential correlation between serum IgA antibodies and mucosal responses during PEDV infection process. Although there is a certain correlation between serum IgG and colostrum IgA antibody levels, this correlation is relatively low (*r* = 0.38) compared to that of serum IgA. Scherba et al. have demonstrated the lack of a relationship between serum IgG antibodies and immunity efficiency against PEDV [25]. Similar observations have also been reported for other enteric viruses, including TGEV [26, 27] and porcine rotavirus (PoRV) [28]. These findings suggest that the serum IgA antibody is a potential substitute for assessing colostrum IgA antibody levels and immunization effects against PEDV, rather than IgG, particularly in the context of oral immunization or refeeding immunization with virulent live PEDV strains. Furthermore, our findings demonstrated that serum IgA antibody levels remained consistently high and stable from 21 days pre-parturition to 14 days postpartum, corroborating the results reported by de Arriba et al. [23]. This observation suggests that serum IgA antibody levels can serve as a predictive indicator for assessing colostrum IgA antibody levels in sows.

## Conclusion

In conclusion, the results presented indicate that serum IgA antibody levels exhibit the strongest correlation with colostrum IgA antibodies, maintaining consistently high and stable concentrations both pre- and post-parturition. These findings suggest that serum IgA levels could serve as a reliable predictor for immunization effects, enabling the assessment of colostrum IgA antibody levels up to 21 days in advance. Consequently, this insight provides veterinarians with the opportunity to adjust or optimize immunization strategies in a timely manner prior to parturition, thereby ensuring the effective transfer of passive immunity to piglets through colostrum postpartum.

## Data Availability

No datasets were generated or analysed during the current study.

## Abbreviations

PED: Porcine Epidemic Diarrhea
PEDV: Porcine Epidemic Diarrhea virus
nAb: Neutralizing antibodies
ASFV: African swine fever virus
ORF: Open reading frames
sIgA: Secretory immunoglobulin A
NC: Negative control
PC: Positive control
DMEM: Dulbecco’s Modified Eagle Medium
CPE: Cytopathic effects
TCID50: 50% tissue culture infective dose
TGEV: Transmissible gastroenteritis virus PoRV porcine rotavirus
